# A novel chemokine‐based signature for prediction of prognosis and therapeutic response in glioma

**DOI:** 10.1111/cns.13944

**Published:** 2022-08-19

**Authors:** Wenhua Fan, Di Wang, Guanzhang Li, Jianbao Xu, Changyuan Ren, Zhiyan Sun, Zhiliang Wang, Wenping Ma, Zheng Zhao, Zhaoshi Bao, Tao Jiang, Ying Zhang

**Affiliations:** ^1^ Department of Neurosurgery, Beijing Tiantan Hospital Capital Medical University Beijing China; ^2^ Department Molecular Neuropathology, Beijing Neurosurgical Institute Capital Medical University Beijing China; ^3^ Chinese Glioma Genome Atlas Network (CGGA) and Asian Glioma Genome Atlas Network (AGGA) Beijing China; ^4^ The Second Affiliated Hospital of Harbin Medical University Harbin China; ^5^ Sanbo Brain Hospital Capital Medical University Beijing China

**Keywords:** chemokine, glioma, immunotherapy, prognostic signature, tumor microenvironment

## Abstract

**Aims:**

Gliomas are the primary malignant brain tumor and characterized as the striking cellular heterogeneity and intricate tumor microenvironment (TME), where chemokines regulate immune cell trafficking by shaping local networks. This study aimed to construct a chemokine‐based gene signature to evaluate the prognosis and therapeutic response in glioma.

**Methods:**

In this study, 1024 patients (699 from TCGA and 325 from CGGA database) with clinicopathological information and mRNA sequencing data were enrolled. A chemokine gene signature was constructed by combining LASSO and SVM‐RFE algorithm. GO, KEGG, and GSVA analyses were performed for function annotations of the chemokine signature. Candidate mRNAs were subsequently verified through qRT‐PCR in an independent cohort including 28 glioma samples. Then, through immunohistochemical staining (IHC), we detected the expression of immunosuppressive markers and explore the role of this gene signature in immunotherapy for glioma. Lastly, the Genomics of Drug Sensitivity in Cancer (GDSC) were leveraged to predict the potential drug related to the gene signature in glioma.

**Results:**

A constructed chemokine gene signature was significantly associated with poorer survival, especially in glioblastoma, *IDH* wildtype. It also played an independent prognostic factor in both datasets. Moreover, biological function annotations of the predictive signature indicated the gene signature was positively associated with immune‐relevant pathways, and the immunosuppressive protein expressions (PD‐L1, IBA1, TMEM119, CD68, CSF1R, and TGFB1) were enriched in the high‐risk group. In an immunotherapy of glioblastoma cohort, we confirmed the chemokine signature showed a good predictor for patients' response. Lastly, we predicted twelve potential agents for glioma patients with higher riskscore.

**Conclusion:**

In all, our results highlighted a potential 4‐chemokine signature for predicting prognosis in glioma and reflected the intricate immune landscape in glioma. It also threw light on integrating tailored risk stratification with precision therapy for glioblastoma.

## Graphical abstract

This study constructed a chemokines‐based gene signature, which was significantly associated with poorer survival, especially in glioblastoma, *IDH* wild‐type subgroup. Due to the gene signature was positively associated with immune‐relevant pathways and immunosuppressive tumor microenvironment state were enriched in the high‐risk group, the chemokines‐based gene signature was considered as a good predictor for patients' immunotherapeutic response.

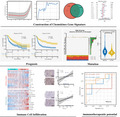

## 1 INTRODUCTION

Glioma is the primary human malignant brain tumor. Up to now, it remains incurable because of the striking genetic and cellular heterogeneity. According to the fifth edition of the WHO classification of tumors of the central nervous system (WHO CNS5),^1^ the median survival time of glioblastoma (GBM, *IDH*‐wildtype) failed to exceed 15 months.^2^, ^3^ Recently, the advancing exploration of molecular mechanism in GBM has paved the way for several emerging therapeutic approaches.^4^, ^5^ For example, with the success of the programmed cell death protein 1 (PD‐1) and programmed cell death 1 ligand 1 (PD‐L1) blockade treatment in melanoma, renal‐cell carcinoma and non‐small‐cell lung cancer,^6^, ^7^, ^8^ immunotherapy represents a new revolutionary strategy in glioma.^9^ To achieve the success of immunotherapy, the tumor microenvironment (TME) plays an indispensable role, where tumor cells and the host immune cell interact. Importantly, chemokines mediate and drive the recruitment of different immune cells in the TME.

Chemokines represents the largest subfamily of cytokines, including four main classes: CC‐, CXC‐, C‐, and CX3C‐chemokines^10^ and 32 members. Massive evidences have shown that chemokines participate in the fundamental physical and pathological processes, such as development, inflammation, infection, and tumorigenesis.^11^ They can not only induce the antitumor immune response by orchestrating T‐cell infiltration to increase interferon‐gamma (IFN‐γ) expression, but also generate the pro‐tumorigenic microenvironment through the recruitment of regulatory T (Treg) cells or tumor‐associated macrophages (TAMs).^12^, ^13^ For example, the overexpression of *CXCL1* had been demonstrated as a poor prognostic indicator and induced radio‐resistance via NF‐κB signaling in glioma patients.^14^ Comprehensive analysis of chemokines has conducted in some solid cancers by leveraging various transcriptomic databases,^15^, ^16^ but it is rarely in gliomas.

Given some studies have characterized the expression profiles and functions of chemokines,^17^ it urgently needs more attention to identify suitable chemokines as therapeutic targets or prognostic biomarkers in gliomas. To address above issues, we systematically investigated the chemokines family in gliomas leveraging an in‐depth and systematic bioinformatics analysis. Due to the indispensable role of this family in controlling glioma TME, we constructed a 4‐chemokine prognostic signature and explored the relationship between this gene signature and the immune‐related landscape as well as the anti‐PD‐1 therapeutic responses. We believed that this robust prognostic signature would improve risk stratification and provide a more optimal and precisive treatment for glioma patients.

## 2 MATERIALS AND METHODS

### 2.1 Samples and datasets

This study retrospectively enrolled RNA sequencing data and corresponding clinical information of 699 glioma patients from The Cancer Genome Atlas (TCGA, https://portal.gdc.cancer.gov/) and 325 glioma patients from Chinese Glioma Genome Atlas (CGGA https://www.cgga.org.cn). All data were normalized by log2 transformed. Somatic mutation and copy number variation (CNV) profiles were obtained from TCGA (https://portal.gdc.cancer.gov/) and analyzed using R package “maftools” and GISTIC 2.0 with a threshold of FDR *Q* < 0.05. The involved clinical characteristics of patients were summarized in Table S1. The dataset of anti‐PD‐1 therapy in glioma were downloaded from the ODC Open Database License (ODbL) (http://opendatacommons.org).

### 2.2 Construction of a chemokine gene signature in glioma

First, preliminary screening was performed to include prognosis‐related genes in TCGA dataset via univariate Cox regression analysis. Next, the least absolute shrinkage and selection operator machine learning algorithm (LASSO) and the SVM‐RFE method were combined to further determine the variables.^18^, ^19^ Lastly, the remained genes were screened through multivariate Cox regression analysis. The riskscore for all patients was determined by taking the sum of regression coefficient for each gene multiplied with its corresponding expression value.

### 2.3 Development and evaluation of the nomogram based on the gene signature

To facilitate the prediction of 1‐, 3‐, and 5‐year overall survival (OS) probability in glioma patients, a nomogram was developed using the “rms” R package. Calibration plots were used to validate the performance of the nomogram using 500 bootstrap resamples.

### 2.4 Biological function and signal pathway analysis

The patients in two datasets were divided into high‐ and low‐risk groups using the median riskscore as a cut‐off. The positive correlated genes with the riskscore were obtained by Pearson correlation analysis (*R* > 0.6, *p* < 0.05) and the ClusterProfiler (R package) was implemented to conduct the gene ontology (GO) analysis.^20^ Then, the Kyoto Encyclopedia of Genes and Genomes (KEGG) and HALLMARK analysis (MSigDB database v7.2) were obtained with the gene set enrichment analysis software (GSEA 4.1.0, http://software.broadinstitute.org/gsea/index.jsp).^21^ The pathways activity scores (*N* = 11) were calculated using PROGENy.^22^

### 2.5 Comprehensive analysis of molecular and immune characteristics

The immune cell infiltration and inflammation activity analysis were obtained with single‐sample gene set enrichment analysis (ssGSEA) method.^23^ The gene set list was from Gabriela and colleagues.^24^ Also, the immune score was evaluated using the ESTIMATE R package, and the immune subtype was identified according to Thorsson and colleagues, including C1 (wound healing), C2 (IFN‐γ dominant), C3 (inflammatory), C4 (lymphocyte depleted), C5 (immunologically quiet), and C6 (TGF‐β dominant).^25^

### 2.6 Immunohistochemistry assay

The formalin‐fixed, paraffin‐embedded glioma tissue was stained according to our previous procedure,^26^ which included 25 glioma samples. Written informed consent was obtained for all patients. Briefly, brain tumor sections were incubated with the PD‐L1 (1:100, ab213524, Abcam, USA), IBA1 (1:100, ab213524, Abcam, USA), TMEM119 (1:100, ab213524, Abcam, USA), CD68 (ZM‐0464, ZSGB‐BIO, China), CSF1R (1:200, 25,949‐1‐AP, Proteintech, USA), and TGFB1 (1:300, 21,898‐1‐AP, Proteintech, USA) antibody overnight at room temperature, respectively. Then, the stained sections were scored by two independent pathologists. The staining intensity was 0–3 points: 0 (negative), 1 (weak), 2 (moderate), and 3 (strong). The extent of staining reflected the percentage of positive cells: 0 (<5%), 1 (6%–25%), 2 (26%–50%), 3 (51%–75%), and 4 (>75%). Staining index was defined as the product of staining intensity and staining extent. Also, PD‐L1 protein expression in The Cancer Proteome Atlas (TCPA, http://tcpaportal.org) was analyzed.

### 2.7 The prognostic analysis and immunotherapy response prediction

To explore the relationship between the gene signature and anti‐PD‐1 immunotherapy, we performed survival analyses in Raul's anti‐PD‐1 treatment dataset.^27^ Moreover, we performed the same analysis for tumor inflammation signature (TIS) and tumor immune dysfunction and exclusion (TIDE, http://tide.dfci.harvard.edu/) score with the timeROC package.^28^

### 2.8 Estimation of drug response in clinical samples

Drug sensitivity was obtained use the R package “pRRophetic.” The predictive model was trained on expression profiles and drug response data of solid cancer cell lines by default 10‐fold cross‐validation. Both datasets provide the estimated concentration for 50% maximal inhibitory concentration (IC50) values as a measure of drug sensitivity.

### 2.9 Quantitative reverse transcription‐polymerase chain reaction (qRT‐PCR)

Total RNA of 28 independent glioma samples were extracted using RNeasy Mini Kit (Qiagen). Written informed consent was obtained for all patients. Then, the RNA intensity was assessed using 2100 Bioanalyzer (Agilent Technologies). The expression levels of each gene were analyzed by ABI 7500 Real‐time PCR System. The relative expression levels of target genes mRNA were obtained by comparative CT method.^29^ The primer sequences used in this study were listed in Table S2.

### 2.10 Statistical analysis

All statistical analyses were performed in R software (version 4.1.1; https://www.r‐project.org/). The Kaplan–Meier method was used to assess the survival time and calculate the difference. Spearman correlation analysis was performed to access the existence of a correlation between variables. Wilcoxon test or Student's *t*‐test was used to compare between two groups for continuous variables. Categorical variables were compared between groups using Chi‐square or Fisher's exact tests. *p* < 0.05 is considered statistically significant.

## 3 RESULTS

### 3.1 Establishment of a chemokine‐related prognostic gene signature

**FIGURE 1:**
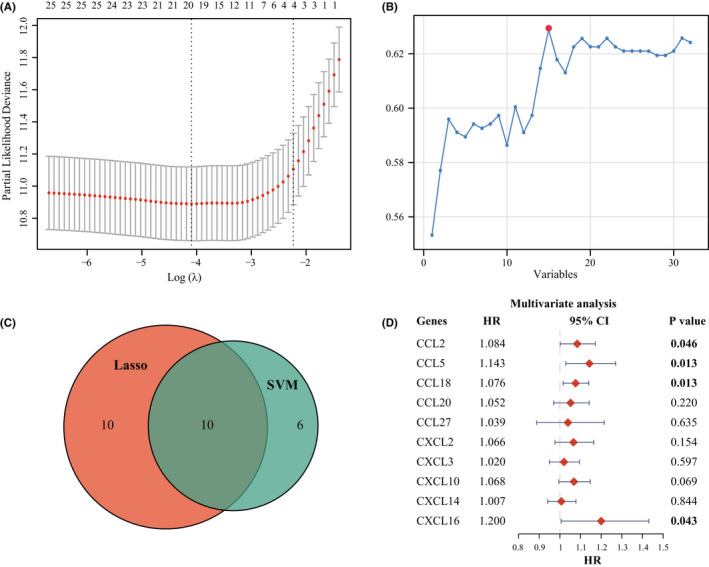
Feature selection of a chemokine‐based gene signature. (A) LASSO coefficient profiles of the remained 27 chemokines. (B) The accuracy of the estimate generation for the SVM‐RFE algorithm. (C) The intersection feature selection between LASSO and SVM‐RFE algorithms. (D) The hazard ratio and *p*‐value of genes involved in multivariate Cox regression in the TCGA dataset

In this study, we enrolled 32 well‐defined chemokine family genes, including 17 CC‐chemokines, 12 CXC‐chemokines, 2 C‐chemokines, and 1 CX3C‐chemokines (Table S3). First, the univariate Cox regression was conducted to identify the 27 prognostic‐related chemokines in TCGA dataset (*p* < 0.05, Table S4). Next, we performed the LASSO algorithm to identify a set of 20 chemokines (Figure 1A) and the SVM‐RFE algorithm to select a set of 16 chemokines (Figure 1B). After intersecting by the LASSO and SVM‐RFE algorithms, 10 chemokines were selected (Figure 1C). Subsequently, the multivariate Cox regression analysis was employed to determine the final candidate genes (*p* < 0.05, Figure 1D). Lastly, four candidate genes and their corresponding cox regression coefficients were used to construct a prognostic index as the formula: Riskscore = 0.0803 × *CCL2* + 0.1336 × *CCL5* + 0.0736 × *CCL18* + 0.1821 × *CXCL16*.

### 3.2 Clinic pathological features related to the gene signature in glioma

**FIGURE 2:**
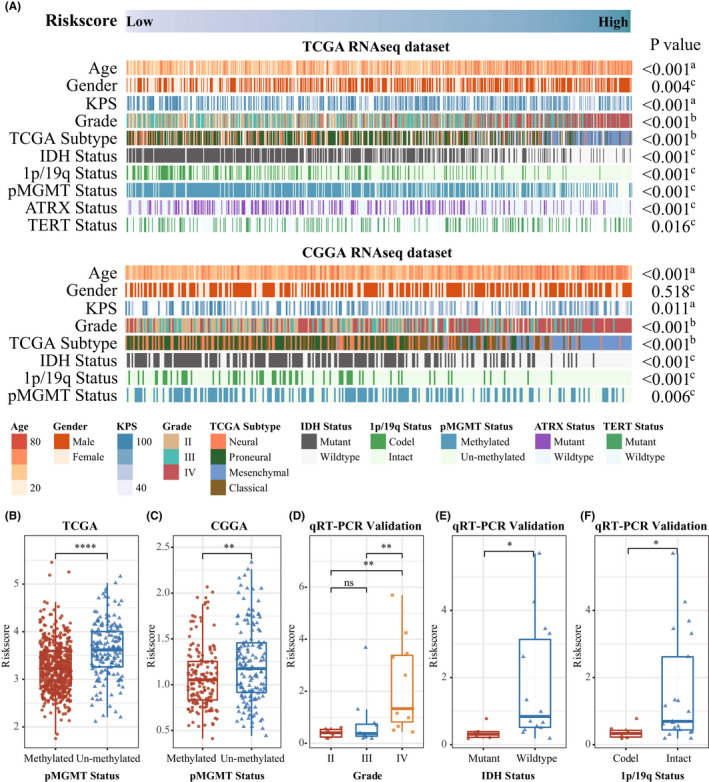
Landscape of clinical and molecular characteristics associated with the gene signature in gliomas. (A) TCGA (top) and CGGA (bottom) were arranged in an increasing order of the 4‐chemokine‐based riskscore. The relationship between the riskscore and patients' clinic pathological characteristics was evaluated (A, Spearman correlation tests; B, One‐way ANOVA test; C, Wilcoxon test). (B and C) The riskscore distribution between *MGMT* promoter methylated group and *MGMT* un‐methylated group in both TCGA (B) and CGGA (C) dataset. (D–F) The riskscore distribution in the different subgroup of glioma in a 28 independent cohort (Wilcoxon test and One‐way ANOVA test)

To investigate the clinical and pathological value of the gene signature, we examined the association between the riskscore and clinic pathological information, including WHO grade, age, gender, TCGA subtype, and some molecular status. As shown in Figure 2A, the patients were ordered by riskscore in both datasets. We found a positive correlation between riskscore and age at diagnosis (all *p* < 0.001), which indicated that younger patients with glioma had lower riskscore. Furthermore, glioma patients with higher riskscore were more likely belong to WHO Grade IV and mesenchymal subtype (all *p* < 0.001), suggesting that the 4‐chemokines‐based signature predict malignant progression. Compared with high‐risk group, *IDH* mutation, 1p/19q codeletion were more occurred in the low‐risk group (all *p* < 0.01). Additionally, the *MGMT* promoter methylation is prognostic and played a powerful predictor of temozolomide sensitivity in gliomas,^2^ we further reanalyze the relationship between riskscore and *MGMT* promoter methylation and found that the higher riskscore are enriched in the *MGMT* promoter un‐methylated group in both datasets (Figure 2B,C). In the TCGA dataset, we found that *ATRX* mutation mainly exists in the low‐risk group (*p* < 0.001), while *TERT* promoter mutation in the high‐risk group (*p* = 0.010). All above results demonstrated that the 4‐chemokine‐based signature is closely related to clinical and pathological features. Then, we reconfirmed the genes expression in an independent 28 glioma samples by qRT‐PCR, and the higher riskscore were founded in the WHO Grade IV (Figure 2D), *IDH*‐wild‐type subgroup (Figure 2E) and 1p19q intact subgroup (Figure 2F), which are consistent with results in TCGA and CGGA datasets.

### 3.3 Prognostic evaluation of 4‐chemokine gene signature

**FIGURE 3:**
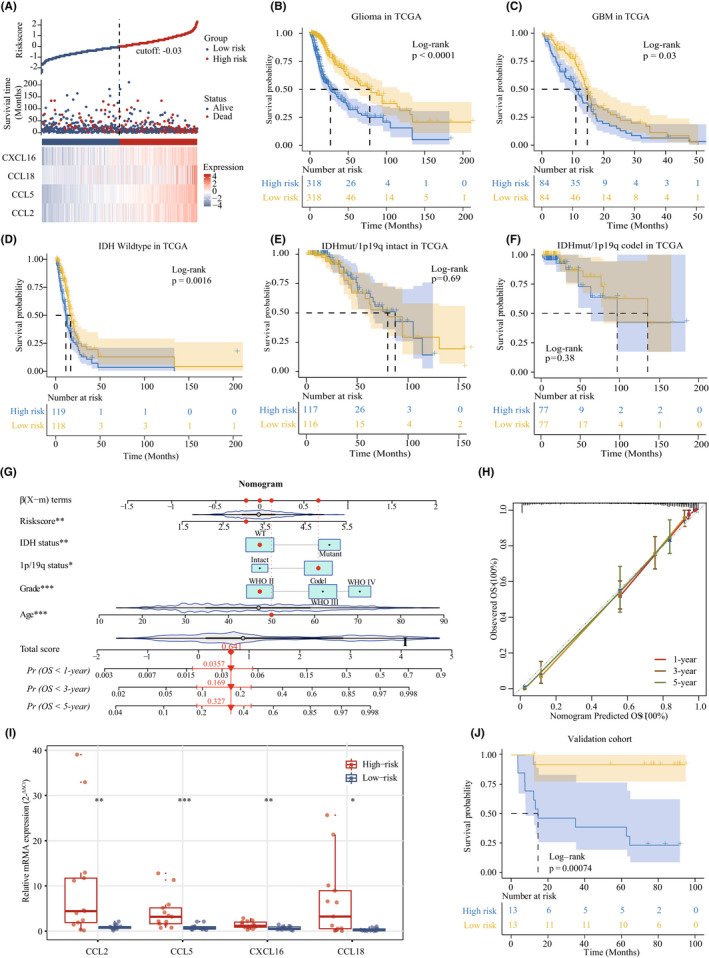
Kaplan–Meier survival analysis stratified by 4‐chemokine gene signature and the nomogram for survival prediction in TCGA dataset. (A) The riskscore of 4‐chemokine‐based signature distribution and survival status distribution for glioma patients. (B–F) Kaplan–Meier survival curves were plotted to estimate the overall survival probabilities in all grade's gliomas (B), GBM (C), *IDH* wildtype (D), *IDH*mut/1p19q intact (E) and *IDH*mut/1p19q codel (F). (G) The nomogram prediction of glioma patients for 1‐, 3‐, and 5‐year OS combining the signatures with clinic pathological features. (H) Calibration curves used to compare the predicted nomogram, the dashed diagonal line represents the ideal nomogram. (I) qRT‐PCR analysis of the four chemokine genes (*CCL2*, *CCL5*, *CCL18*, and *CXCL16*) expression between high‐ and low‐risk group in an independent validation cohort, 18S was used as an internal reference. (J) Kaplan–Meier survival curves were plotted to estimate the overall survival probabilities in an independent validation gliomas group

To further evaluate the relationship between the gene signature and patients' survival time, the four gene expression details and the patients' survival status are ranked by riskscore values, and all patients were assigned to high‐risk or low‐risk groups based on the median cut‐off point (−0.03) in TCGA dataset (Figure 3A **)**. The patients in the low‐risk group had a significantly better prognosis than that in the high‐risk group (log‐rank test, *p* < 0.0001) (Figure 3B). For GBM (WHO Grade IV), patients in the high‐risk group are associated with significantly shorter survival time (log‐rank test, *p* = 0.03, Figure 3C). According to the WHO CNS5, we found that the survival probability of the high‐risk patients decreased significantly in the *IDH* wild‐type subgroup (log‐rank test, *p* = 0.0016, Figure 3D). When we further explored the prognostic role of the signature in the patients with *IDH* mutant, we found that there is no difference (Figure 3E,F), which confirmed the important role of *IDH* status in gliomas.^30^ The above‐mentioned results are well validated in independent CGGA dataset (Figure S1A–F). Notably, the riskscore was confirmed as an independent prognostic factor for overall survival of glioma patients in both datasets (Tables S5 and S6). To improve the feasibility of clinical application in the individual glioma patients, we generated a nomogram that integrates independent prognostic factors to predict the 1‐, 3‐, and 5‐year overall survival rates, and the red arrow shows an example (Figure 3G and Figure S1G). The calibration plots are extremely close to an ideal model (Figure 3H and Figure S1H), indicating the nomogram possessed better predictive power and facilitate the clinical decision‐making. Importantly, we again verified the expression of four chemokine genes by qRT‐PCR method in an independent cohort including 28 glioma samples (Figure 3I), notably, the riskscore also predicted the prognosis of glioma patients well (Figure 3J).

### 3.4 Comprehensive analyses of genomic alterations

**FIGURE 4:**
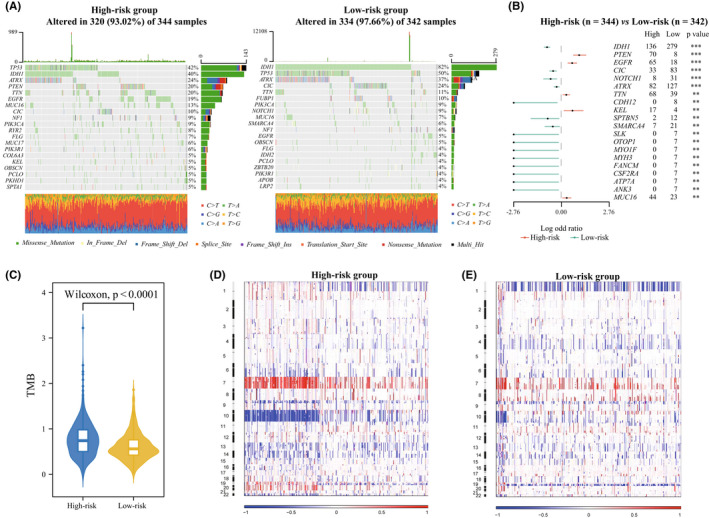
Distinct genomic alterations between high‐ and low‐risk group. (A) Differential somatic mutations were detected between high‐ and low‐risk group. (B) Top 20 significantly differential mutational genes (Fisher's exact test). (C) TMB between high‐ and low‐risk group (Wilcoxon test). (D and E) Distinct CNA profiles between gliomas in high‐ and low‐risk groups

To unveil the association between genomic features and the gene signature, TCGA samples with available mutation and copy number variation (CNA) information were collected. According to stratified group above mentioned, the top frequent mutations contain *IDH1*, *TP53*, *ATRX*, *CIC*, *TTN*, *MUC16*, *NOTCH1*, *EGFR*, *NF1*, and *PIK3CA*. While 82% of cases in low‐risk group carried *IDH1* mutation (Figure 4A), which represented an earlier driven mutation in the glioma and indicated a better prognostic outcome.^30^ 20% patients carried the *PTEN* mutation in the high‐risk group (Figure 4A), Zhao et al had demonstrated that GBM with *PTEN* mutation induce more immunosuppressive TME and is resistant to anti‐PD‐1 therapy.^27^ Moreover, the *EGFR* and *MUC16* mutations were also significantly enriched in cases within high‐risk group, while mutations in *CIC*, *NOTCH1*, *ATRX*, and *CDH12* occurred more frequently in the low‐risk group (Figure 4B), these characteristics are consistent with the update WHO CNS5 classification of glioma. We also observed that significantly different mutation frequencies in *SLK*, *OTOP1*, *MYO1F*, *MYH3*, *FANCM*, *CSF2RA*, *ATP7A*, and *ANK3*, while further studies were needed to explore their roles in glioma. Additionally, we confirmed that there was more tumor mutation burden (TMB) accumulated in the high‐risk group (*p* < 0.0001, Figure 4C), which implied the more tumor heterogeneity and chemotherapy resistance.^31^ Subsequently, CNA data were employed to explore distinct chromosomal alteration. Notably, Chr 7 amplification paired with Chr 10 loss, a representative characteristic in GBM,^32^ were also enriched in the high‐risk group (Figure 4D). However, the incidence of the 1p/19q codeletion, which is a genomic hallmark in oligodendroglioma,^33^ was higher in the low‐risk group (Figure 4E). These results also are verified in our independent cohort in vitro (Figure 2D).

### 3.5 Biological processes and signal pathways analysis

**FIGURE 5:**
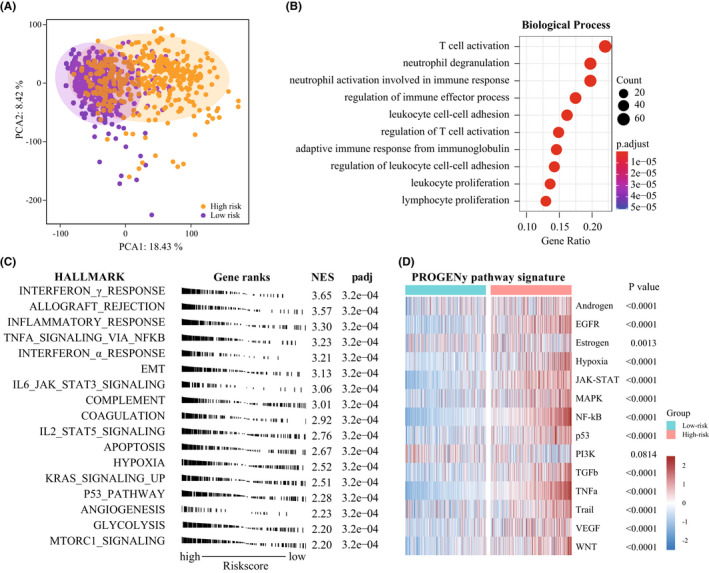
Biological processes and signal pathways associated with the 4‐chemokine signature in TCGA dataset. (A) Correlation between chemokines‐based prognostic signature and transcriptomic expression profiles. (B) Biological processes enrichment of positively associated genes in TCGA dataset. (C) Enriched gene sets in HALLMARK collection in TCGA dataset. (D) Heatmap of signaling pathway activity scores by PROGENy (Wilcoxon test)

To explore the biological functions and pathways associated with 4‐chemokine‐based signature, the gene ontology (GO), GSEA, and PROGENy pathway analysis were performed. First, we carried out principal component analysis to explore the transcriptomic features associated with the gene signature, which showed a strong association between whole transcriptome expression profile and riskscore (Figure 5A and Figure S2A), implying distinct biological characteristics between two groups. Afterward, we screened the genes that positively correlated with riskscore (Pearson, *R* > 0.6, *p* < 0.05), 900 and 631 genes were separately identified in TCGA and CGGA datasets, respectively. Then, GO analysis showed that the positively associated genes were mainly immune‐relevant, such as T‐cell activation, neutrophil degranulation, neutrophil activation, and regulation of immune effector process in both databases (Figure 5B and Figure S2B). Additionally, the hallmark analysis also showed that the gene signature was not only closely enriched the interferon γ response, but also associated with epithelial‐mesenchymal transition (EMT), apoptosis, hypoxia and angiogenesis (Figure 5C and Figure S2C), suggesting that our gene signature could also predict malignant process in glioma. Lastly, NF‐κB, MAPK, TNFα, and WNT signaling pathway, especially for JAK–STAT, TGFβ pathways were obviously enriched in the high‐risk group (Figure 5D and Figure S2D).

### 3.6 Immune cell infiltration and inflammatory profiles related to the gene signature

**FIGURE 6:**
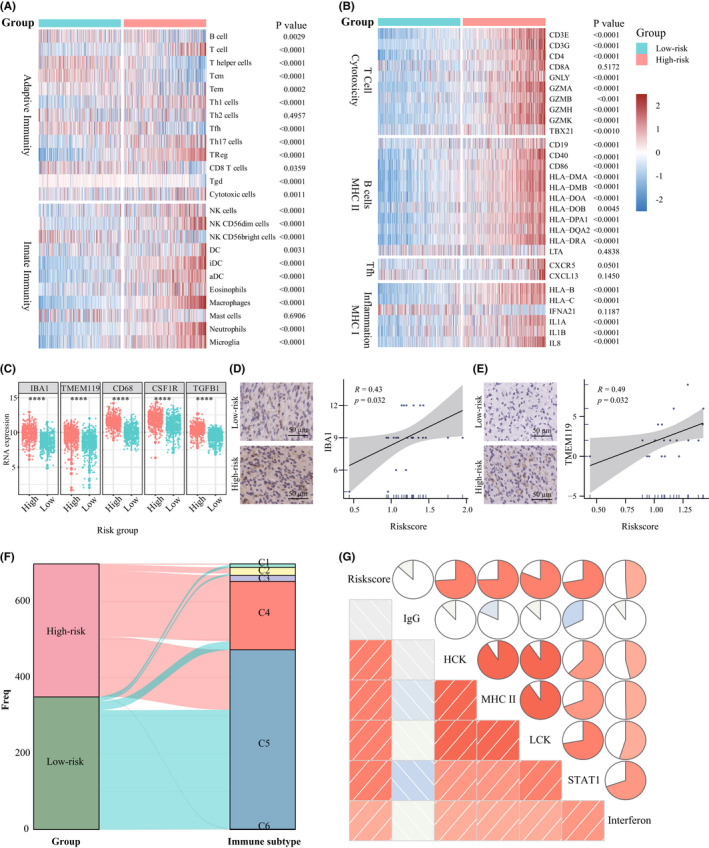
Immune cell infiltration and inflammatory profiles of the gene signature in TCGA dataset. (A) Heatmap of adaptive and innate immune cell types in high‐ and low‐risk group. (B) Heatmap of the MHC‐, costimulation‐, and inflammatory‐related genes expression in glioma patients from high‐ and low‐risk group. (C) The representative GAM related gene expression level between high‐ and low‐risk group in the TCGA dataset. (D) The representative IHC images of IBA1 and the correlation plot between riskscore and IBA1 protein expression. (E) The representative IHC images of TMEM119 and the correlation plot between riskscore and TMEM119 protein expression. (F) Sankey plot shows the relationship between glioma patients stratified by riskscore and 6 immune subtypes defiend by Thorsson et al. (G) The relationship between riskscore and inflammatory activity in glioma. (Wilcoxon test)

Inspiring by the tight relationship between the 4‐chemokine signature and immune‐related biological processes and pathways, we elaborately investigated both innate immune cells and adaptive immune cells infiltration.^24^ Comparing with the low‐risk group, the high‐risk patients induced a higher abundance of DCs, Tregs, macrophages, neutrophils, and microglia as well as lower abundance of NK CD56 bright cells (Figure 6A and Figure S3A), which conferred suppressive TME.^34^ Furthermore, patients in the high‐risk group had an eminent increment of expression level related to MHC II, B‐cell costimulation, cytotoxic T cells, and MHC I inflammation‐related genes (Figure 6B and Figure S3B). These higher adaptive immune gene markers represent a fundamental feature of the host defense against tumor development.^24^ The paradoxical phenomenon in the TME makes us speculated that the high‐risk patients retain their antitumor immunity potentiality, which might be blocked by the suppressive TME. Subsequently, we further evaluated the representative glioma‐associated microglia/macrophage (GAM)‐related genes expression level between high‐ and low‐risk group,^35^ higher expression level of *IBA1*, *TMEM119*, *CD68*, *CSF1R*, and *TGFB1* was found in the high‐risk group (Figure 6C and Figure S3C). Our in vitro glioma IHC analysis also confirmed the riskscore positively correlated with IBA1, TMEM119, CD68, CSF1R, and TGFB1 protein expression (Figure 6D,E and Figure S3D–F). Then, we compare our signature with other immune subtypes derived from pan‐cancer,^25^ the high‐risk group contains C2 (IFN‐γ dominant), C3 (inflammatory), C4 (lymphocyte depleted), and C5 (immunologically quiet) immune subtypes, but nearly all cases in the low‐risk group were assigned to C5 subtype, which is mainly characterized with less TAMs (Figure 6F and Figure S3G). Lastly, we analyzed the activated inflammatory activity profile. Surprisingly, the riskscore was positively associated with HCK, LCK, STAT1, interferon and MHC II profile, but was negatively associated with IgG, suggesting that high‐risk patients presented a more activated inflammatory level (Figure 6G and Figure S3H). These findings confirmed the important immune function related to the gene signature and the high‐risk patients retained a more activated inflammatory state but more suppressive TME.

### 3.7 The immunotherapy potentiality with the 4‐chemokine gene signature

**FIGURE 7:**
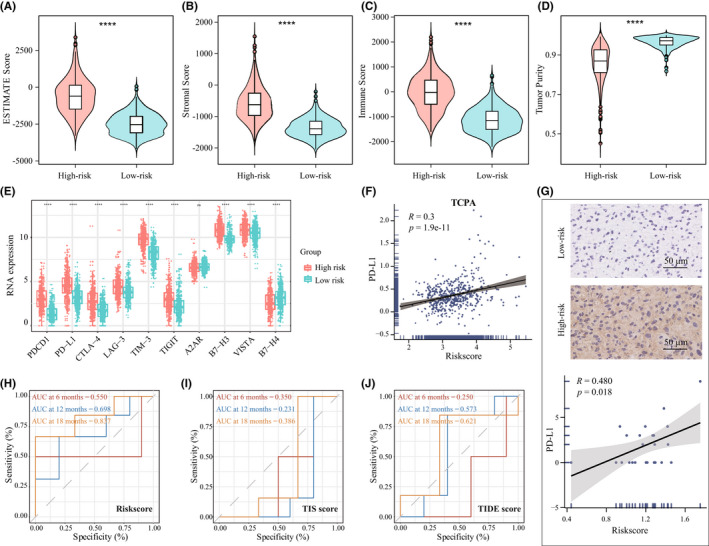
Distribution of immune‐response markers and the prognostic value of riskscore in patients with anti‐PD‐1 therapy. (A–D) Distribution of estimate score, stroma score, immune score and tumor purity in the TCGA dataset. (E) Distribution of different immune checkpoint mRNA expression in high‐ and low‐risk groups. (F) The correlation of riskscore and PD‐L1 protein expression in TCPA. (G) The distribution of PD‐L1 protein expression in high‐ and low‐risk groups by IHC staining. Scale bar, 50 μm. (H–J) ROC analysis of riskscore (H), TIS (I), and TIDE (J) score on overall survival at 6‐, 12‐, and 18‐month follow‐up in GBM patients receiving anti‐PD‐1 therapy

Recently, immunotherapy is emerging to revolute the classical treatment.^36^ To assess the potential role of the riskscore in clinical immunotherapy for glioma, we further assessed the glioma TME characteristics via ESTIMATE algorithm.^37^ The results showed that the higher estimate score was found in high‐risk group (Figure 7A), and the immune and stromal scores were also higher in high‐risk group (Figure 7B,C), while the low‐risk group possessed higher tumor purity (Figure 7D), which predicts a better prognosis.^34^ Next, to further elucidate the underlying immunotherapeutic potential, the expression of different immune checkpoint markers was compared.^38^ As expected, excluding Adenosine A2a Receptor (A2AR), high‐risk group had a significantly higher immune checkpoint genes expression level than low‐risk group (Figure 7E), which represented a higher immune evasion. Meanwhile, the riskscore showed a positive correlation with PD‐L1 protein expression in TCPA database (Figure 7F, *R* = 0.3, *p* = 1.9e‐11). Also, our own glioma tissue IHC analysis confirmed the positive correlation (Figure 7G, *R* = 0.66, *p* = 0.01, *n* = 24). Finally, in GBM patients receiving anti‐PD‐1 therapy,^27^ the AUCs of our riskscore in predicting overall survival from anti‐PD‐1 therapy was better (Figure 7H) compared to the T‐cell‐inflamed signature (TIS, Figure 7I)^28^ and the TIDE score (Figure 7J) at 6‐, 12‐, and 18‐month follow‐up point. Collectively, these results indicated that the riskscore of our 4‐chemokine signature showed a good predictor for patients' response to anti‐PD‐1 therapy.

### 3.8 Identification of potential therapeutic agents for high‐risk group patients

**FIGURE 8:**
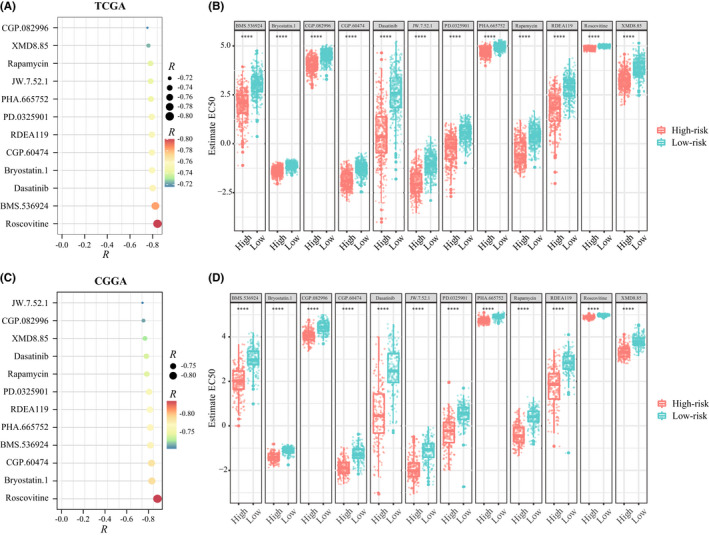
Identification of candidate agents with higher drug sensitivity in high‐risk group patients. (A and C) The dot chart showed the correlation coefficient between riskscore and the estimate half maximal inhibitory concentration (IC50) of 12 candidate drugs in TCGA (A) and CGGA (C) datasets. (B and D) The estimated IC50 of 12 candidate drugs were compared between high‐ and low‐risk groups in TCGA (B) and CGGA (D) datasets (Wilcoxon test)

Given that chemotherapy remain a common adjunctive approach *in clinic*, we explored candidate agents with higher drug sensitivity in high‐risk patients. Through training a predictive model with the cell line data derived from the Genomics of Drug Sensitivity in Cancer (GDSC), the R package “pRRophetic” was used to estimate the chemotherapeutic sensitivity in TCGA and CGGA datasets. First, compounds with a negative correlation between the estimated IC50 value and the riskscore were selected (Spearman, *R* < −0.80, *p* < 0.05). After intersecting results in TCGA and CGGA datasets, twelve compounds were obtained, including Roscovitine, Bryostatin.1, CGP.60474, BMS.536924, PHA.665752, RDEA119, PD.0325901, Rapamycin, Dasatinib, XMD8.85, CGP.082996, and JW.7.52.1 (Figure 8A,C). Further analyses showed that all these compounds had lower estimated IC50 values in high‐risk group (Figure 8B,D). It indicated that these agents were promising therapeutic drugs for high‐risk patients with glioma.

## 4 DISCUSSION

Chemokines regulate cancer‐related inflammation, tumor microenvironment, tumor growth, and metastasis.^39^ Recently, chemokine‐based risk signatures have showed a good prediction for clinical outcome and immunotherapy response in lung adenocarcinoma and pancreatic adenocarcinoma.^40^, ^41^ Additionally, a dysregulated chemokine network is one of the characteristics of glioma.^39^ Thus, we performed the comprehensive analysis of the chemokine genes in glioma. The constructed 4‐chemokine‐based signature was confirmed to be associated with dismal prognosis and was an independent risk factor. Meanwhile, the immune landscape and inflammatory profile were explored, patients in the high‐risk group had significantly higher T‐cell dysfunction markers and PD‐L1 expression level, indicating the obvious inhibitory TME characteristic. Lastly, 12 compounds were identified as potential therapeutic agents for high‐risk glioma patients.

In the previous study, it had been demonstrated that *CXCL1* played a poor prognostic indicator and induced radio‐resistance via NF‐κB signaling in glioma patients.^14^ Also, Feng et al reported that beta‐2 microglobulin (B2M) was highly correlated with two chemokines (*CXCL10* and *CCL5*) and mediates GAM infiltration via these two chemokines in glioma.^42^ In the other chemokine genes, CCL2 secreted by glioma cells promotes tumor growth and migration, its expression is correlated with GAMs accumulation in GBM.^43^ Additionally, CCL2 blockade reduced GAMs infiltration and prolonged the survival of C57BL/6 mice bearing GL261 glioma.^44^ Then, CCL5/CCR5 axis induces proliferation and invasion in GBM via calcium‐dependent matrix metalloproteinase 2,^45^ and regulates chemoresistance of temozolomide in GBM.^46^ And, CCL18 enhances the invasion and proliferation of U251 glioma cells.^47^ Moreover, CXCL16 also promotes GBM growth and migration, also drives GAMs polarization.^48^ All four chemokines are included in our constructed gene signature, further suggesting that the gene signature could play an indispensable role in modulating glioma TME and predict poorer prognosis. The higher riskscore are positively related to malignant clinical pathological characteristics, including high WHO grade, mesenchymal subtype, *IDH* wildtype, and *MGMT* promoter unmethylated. Also, the patients exhibit a shorter survival time in the high‐risk group, especially for patients with GBM with *IDH* wild‐type subgroup. Additionally, the gene signature plays an independent prognostic factor. A nomogram that integrates the clinicopathological features and riskscore showed a good accuracy. Collectively, these results indicated that the 4‐chemokine signature has potential clinical application in the future.

Second, the high‐risk patients showed an enrichment of immune‐related processes and pathways, which may be the main reason for the difference in survival time of glioma patients. Then, our results confirmed that patients in the high‐risk group not only presented an inflammatory activation state, but also enriched a higher abundance of GAMs and more adaptive immune gene markers. These classical immune regulation pathways imply the complexity of the glioma TME ecosystem Notably, low‐risk patients featured lower infiltration of GAMs and mainly in an immunologically quiet subtype, indicating that these patients may show a better outcome.^25^ In the future, further biological experimental verification is needed to clarify these bioinformatics analyses.

Third, the interesting finding is the application of 4‐chemokine signature in therapeutic prediction. We found that high‐risk patients presented higher level of TMB and PD‐L1 expression, as well as the activated MAPK pathway. Arrieta et al found that MAPK/ERK pathway activation in recurrent GBM patients is predictive of response to PD‐1 blockade.^49^ The immune infiltration analysis showed that more innate and adaptive immune factors occurred in the high‐risk group patients, which represents a fundamental potentiality of the host defense against tumor and these patients could benefit from immunotherapy. However, the higher inhibitory immune cell infiltration indicated a state of immunosuppression in the high‐risk patients. These paradoxical features indicated that once reversing the inhibitory TME, the per se antitumor ability would exert more effectively. Thus, we speculated that the high‐risk patients would induce effectively antitumor immune response with immunotherapy, which were verified in the survival analysis of anti‐PD‐1 therapy in GBM. As shown in Figure 7H,I, our gene signature exhibited a good predictor for patients' response in different follow‐up point. Collectively, these results suggest that the high‐risk patients may be response to ICI therapy.

Lastly, 12 compounds have been obtained as promising therapeutic drugs for high‐risk patients with glioma. Through comprehensive literature mining, we found that these drugs are inhibitors for CDK, PKC, c‐MET, MEK, ERK, and MTOR, which is consistent with the high enrichment of MAPK, WNT, and TNFα signaling pathways in the high‐risk patients. The experimental and clinical evidence of these candidate compounds were listed in Table S7. Among them, Dasatinib, Rapamycin, and Mirdametinib (PD.0325901) have been explored in silico or in vitro assay, and some have entered clinical trial phase. However, further experiments are needed to verify.

## AUTHOR CONTRIBUTIONS

WF, YZ, and TJ contributed to conception and design of the study. WF, WM, ZZ, and CR organized the database. WF, GL, ZW, ZS, ZB, and YZ contributed to analysis and interpretation of data. WF, JX, and DW perform the experiments. WF and YZ contributed to the first draft of the manuscript. WF, TJ, and YZ revise the manuscript. All authors contributed to the article and approved the submitted version.

## FUNDING INFORMATION

This study was supported by funds from the National Natural Science Foundation of China (81972337, 81761168038); the public welfare development and reform pilot project of Beijing Medical Research Institute (JYY 2019–5); the CAMS Innovation Fund for Medical Sciences (2019‐I2M‐5‐021), Beijing Municipal Administration of Hospitals' Mission Plan (SML20180501); the China Postdoctoral Science Foundation (2021M702308, 2022M712218); Beijing Postdoctoral Research Foundation (2021‐ZZ‐022) and Natural Science Foundation of Beijing Municipality (JQ20030, 7222018).

## CONFLICT OF INTEREST

The authors have no conflict of interest.

## Supporting information


Appendix S1

## Data Availability

All data generated and analyzed in this study are included in this published article and additional files." cd_value_code="text
