# Supplementary material for: A novel chemokine‐based signature for prediction of prognosis and therapeutic response in glioma
Source: CNS Neurosci Ther. 2022 Aug 19;28(12):2090–103. doi: 10.1111/cns.13944 (PMC9627384; doi:10.1111/cns.13944)
Supplement: Supplementary file 1 — Appendix S1 [file CNS-28-2090-s001.docx]

# Supporting information

## Supplementary Tables

**Supplement Table 1 Clinical characteristics of samples in two datasets**

| **Variable** | **Training dataset**  **(TCGA N = 699*^1^*)** | | **Validation dataset (CGGA N = 325*^1^*)** | |
| --- | --- | --- | --- | --- |
| **Age** | 47 (15) | | 43 (12) | |
| **Grade** |  | |  | |
| WHO II | 223 (35%) | | 103 (32%) | |
| WHO III | 245 (39%) | | 79 (25%) | |
| WHO IV | 168 (26%) | | 139 (43%) | |
| **Gender** |  | |  | |
| Female | 268 (42%) | | 122 (38%) | |
| Male | 368 (58%) | | 203 (62%) | |
| **1p/19q status** |  | |  | |
| Codel | 172 (25%) | | 64 (20%) | |
| Intact | 520 (75%) | | 261 (80%) | |
| **IDH status** |  | |  | |
| Mutant | 443 (64%) | | 175 (54%) | |
| Wildtype | 246 (36%) | | 149 (46%) | |
| **MGMT promoter status** | |  | |  |
| Methylated | 492 (75%) | | 157 (51%) | |
| Unmethylated | 168 (25%) | | 149 (49%) | |
| **TCGA Subtype** |  | |  | |
| Classical | 92 (16%) | | 74 (23%) | |
| Mesenchymal | 105 (19%) | | 68 (21%) | |
| Neural | 115 (20%) | | 81 (25%) | |
| Proneural | 250 (44%) | | 102 (31%) | |
| **Radiotherapy**  Yes  No |  | | 244 (79%)  66 (21.3%) | |
| **Chemotherapy**  Yes  No |  | | 193 (63%)  111 (36.5%) | |
| **TERT promoter status** |  | |  | |
| Mutant | 166 (48%) | |  | |
| Wildtype | 181 (52%) | |  | |
| **ATRX** |  | |  | |
| Mutant | 206 (30%) | |  | |
| Wildtype | 477 (70%) | |  | |
| **KPS*^2^*** | 80 (9) | | 80 (14) | |
| ***^1^*** Mean (SD); n (%); ***^2^*** Karnofsky Performance Status  **Supplement Table 2 Primer of *CCL2*, *CCL5*, *CCL18*, *CXCL16* and *18S* gene**   \| Gene Name \| Sequence \| \| --- \| --- \| \| CCL2 \| Forward-5′CTCTCGCCTCCAGCATGAAA3′ \| \|  \| Reverse-5′CACACTTGGCGGTTCTTTCG3′ \| \| CCL5 \| Forward-5′GGATCAAGACAGCACGTGGA3′ \| \|  \| Reverse-5′CACACTTGGCGGTTCTTTCG3′ \| \| CCL18 \| Forward-5′CTGCTGCCTCGTCTATACCTC3′ \| \|  \| Reverse-5′GGCATAGCAGATGGGACTCT3′ \| \| CXCL16 \| Forward-5′AATTTCTTCCGACTCCCCGC3′ \| \|  \| Reverse-5′CCACAATCCCCGAGTAAGCA3′ \| \| 18S \| Forward-5′AGAAACGGCTACCACATCCA3′ \| \|  \| Reverse-5′CCCTCCAATGGATCCTCGTT3′ \| | | | |  |

**Supplement Table 3 The details of chemokine family members**

| **Name** | | **Synonym(s)** | **Amino Acids** | **Ligand Location** | **Molecular Weight (Da)** | **Chemokine Receptor(s)** | **Receptor Location** |
| --- | --- | --- | --- | --- | --- | --- | --- |
| **C Chemokines** | |  |  |  |  |  |  |
| XCL1 | | lymphoactin a, SCM-1a | 114 | 1q21-q25 | 12517 | XCR1 | 3p21 |
| XCL2 | | lymphoactin b, SCM-1b | 114 | 1q23 | 12567 | XCR1 | 3p21 |
| **CC Chemokines** | |  |  |  |  |  |  |
| CCL2 | | MCP-1, MCAF | 99 | 17q11.2-q12 | 11025 | CCR2 | 3p21 |
| CCL3 | | MIP-1α, LD78α | 92 | 17q11-q21 | 10085 | CCR1, CCR5 | 3p21 |
| CCL4 | | MIP-1β, LAG-1, ACT-2 | 92 | 17q11-q23 | 10212 | CCR5 | 3p21 |
| CCL5 | | RANTES | 91 | 17q11.2-q12 | 9990 | CCR1, CCR3, CCR5 | 3p21 |
| CCL8 | | MCP-2 | 99 | 17q11.2 | 11246 | CCR3 | 3p21 |
| CCL13 | | MCP-4 | 98 | 17q11.2 | 10986 | CCR2, CCR3 | 3p21 |
| CCL14 | | HCC-1 | 93 | 17q11.2 | 10678 | CCR1 | 3p21 |
| CCL17 | | TARC | 94 | 16q13 | 10507 | CCR4 | 3p24 |
| CCL18 | | DC-CK1, PARC, MIP-4 | 89 | 17q11.2 | 9849 | ? |  |
| CCL19 | | MIP-3β, ELC, exodus-3 | 98 | 9p13 | 10993 | CCR7 | 17q12-q21.1 |
| CCL20 | | MIP-3α, LARC, exodus-1 | 96 | 2q33-q37 | 10762 | CCR6 | 6q27 |
| CCL21 | | 6Ckine, SLC, exodus-2 | 134 | 9p13 | 14646 | CCR7 | 17q12-q21.2 |
| CCL22 | | MDC, STCP-1 | 93 | 16q13 | 10580 | CCR4 | 3p22 |
| CCL25 | | TECK, MIP-4a | 150 | 19p13.2 | 16639 | CCR9 | 3p21 |
| CCL26 | | eotaxin-3 | 94 | 7q11.2 | 10648 | CCR3 | 3p21 |
| CCL27 | | Eskine, CTACK, ILC | 112 | 9p13 | 12618 | CCR10 | 3p21 |
| CCL28 | | SCYA28, MEC, CCK1 | 127 | 5p12 | 14280 | CCR3, CCR10 | 3p21 |
| **CXC Chemokines** | |  |  |  |  |  |  |
| CXCL1 | | GROa, MGSA-a^+^ | 107 | 4q21 | 11301 | CXCR1, CXCR2 | 2q35 |
| CXCL2 | | GROb, MGSA-b, MIP-2a^+^ | 107 | 4q21 | 11389 | CXCR2 | 2q35 |
| CXCL3 | | GROg, MGSA-g, MIP-2b^+^ | 107 | 4q21 | 11342 | CXCR2 | 2q35 |
| CXCL5 | | ENA-78^+^ | 114 | 4q13-q21 | 11972 | CXCR2 | 2q35 |
| CXCL6 | | GCP-2^+^ | 114 | 4q21 | 11897 | CXCR1, CXCR2 | 2q35 |
| CXCL9 | | Mig | 125 | 4q21 | 14019 | CXCR3 | Xq13 |
| CXCL10 | | IP-10 | 98 | 4q21 | 10856 | CXCR3 | Xq13 |
| CXCL11 | | I-TAC | 94 | 4q21.2 | 10365 | CXCR3 | Xq13 |
| CXCL12 | | SDF-1α/β | 93 | 10q11.1 | 10666 | CXCR4 | 2q21 |
| CXCL13 | | BLC, BCA-1 | 109 | 4q21 | 12664 | CXCR5 | 11q23.3 |
| CXCL14 | | BRAK | 99 | 5q31 | 11722 | ? |  |
| CXCL16 | | SRPSOX, SCYB16 | 254 | 17p13 | 27579 | CXCR6 | 3p21 |
| **CX3C Chemokines** | | |  |  |  |  |  |
| CX3CL1 | fractalkine | | 397 | 16q13 | 42202 | CX3CR1 | 3p21 |

**Supplement Table 4 Univariate analysis of chemokines in TCGA dataset**

| **Gene** | **HR***^1^* | **95% CI***^1^* | | **P value** |
| --- | --- | --- | --- | --- |
|  |  | **Low** | **High** |  |
| CXCL10 | 1.37 | 1.30 | 1.44 | 3.74E-32 |
| CCL20 | 1.39 | 1.31 | 1.47 | 4.80E-29 |
| CXCL11 | 1.38 | 1.30 | 1.47 | 1.00E-26 |
| CXCL9 | 1.34 | 1.26 | 1.42 | 1.75E-22 |
| CCL5 | 1.51 | 1.39 | 1.65 | 8.21E-22 |
| CCL27 | 1.91 | 1.67 | 2.19 | 5.75E-21 |
| XCL2 | 1.69 | 1.49 | 1.91 | 2.31E-16 |
| XCL1 | 1.70 | 1.49 | 1.93 | 3.02E-16 |
| CXCL6 | 1.28 | 1.21 | 1.36 | 5.17E-16 |
| CCL2 | 1.32 | 1.23 | 1.41 | 1.95E-15 |
| CCL13 | 1.33 | 1.24 | 1.42 | 3.70E-15 |
| CCL26 | 1.40 | 1.28 | 1.53 | 2.08E-13 |
| CXCL16 | 1.87 | 1.56 | 2.24 | 8.65E-12 |
| CXCL14 | 1.22 | 1.15 | 1.30 | 5.03E-10 |
| CX3CL1 | 0.67 | 0.59 | 0.77 | 1.68E-09 |
| CCL18 | 1.16 | 1.10 | 1.22 | 1.78E-09 |
| CCL8 | 1.21 | 1.14 | 1.29 | 1.95E-09 |
| CXCL1 | 1.20 | 1.13 | 1.27 | 6.31E-09 |
| CCL19 | 0.84 | 0.78 | 0.90 | 3.18E-06 |
| CXCL3 | 1.18 | 1.10 | 1.27 | 4.10E-06 |
| CXCL13 | 1.14 | 1.07 | 1.21 | 3.19E-05 |
| CCL25 | 1.24 | 1.11 | 1.38 | 7.80E-05 |
| CCL17 | 1.18 | 1.07 | 1.30 | 1.13E-03 |
| CCL21 | 0.75 | 0.63 | 0.89 | 1.25E-03 |
| CCL22 | 1.17 | 1.06 | 1.30 | 2.73E-03 |
| CCL3 | 0.93 | 0.88 | 0.99 | 0.02 |
| CXCL2 | 1.09 | 1.00 | 1.18 | 0.05 |
| CXCL12 | 0.88 | 0.77 | 1.01 | 0.07 |
| CCL4 | 0.95 | 0.90 | 1.01 | 0.11 |
| CCL14 | 1.06 | 0.97 | 1.15 | 0.20 |
| CCL28 | 1.07 | 0.95 | 1.19 | 0.27 |
| CXCL5 | 1.03 | 0.96 | 1.10 | 0.44 |

***^1^*** HR = Hazard Ratio, ***^2^***CI = Confidence Interval

**Supplement Table 5 Univariate and multivariate analysis of clinical prognostic parameters in TCGA dataset**

| **Variable** | **Univariate analysis** | | | **Multivariate analysis** | | |
| --- | --- | --- | --- | --- | --- | --- |
|  | **HR***^1^* | **95% CI***^1^* | **P value** | **HR***^1^* | **95% CI***^1^* | **P value** |
| Age | 1.07 | 1.06-1.08 | **< 0.001** | 1.06 | 1.05-1.08 | **< 0.001** |
| Gender | 1.11 | 0.84-1.46 | 0.5 |  |  |  |
| Grade |  |  |  |  |  |  |
| WHO II | - | - |  | - | - |  |
| WHO III | 3.06 | 1.93-4.83 | **< 0.001** | 2.06 | 1.27-3.35 | **0.003** |
| WHO IV | 19.1 | 12.0-30.4 | **< 0.001** | 3.01 | 1.61-5.62 | **< 0.001** |
| IDH status | 8.61 | 6.26-11.8 | **< 0.001** | 1.88 | 1.09-3.23 | **0.023** |
| 1p/19q status | 4.42 | 2.68-7.27 | **< 0.001** | 2.04 | 1.14-3.65 | **0.017** |
| MGMT promoter status | 2.97 | 2.18-4.06 | **< 0.001** | 1.19 | 0.82-1.74 | 0.4 |
| Riskscore | 3.52 | 2.77-4.46 | **< 0.001** | 1.75 | 1.26-2.44 | **< 0.001** |
| *^1^*HR = Hazard Ratio, ***^2^***CI = Confidence Interval | | | | | | |

| **Supplement table 6 Univariate and multivariate analysis of clinical prognostic parameters in CGGA dataset**   \| **Variable** \| **Univariate analysis** \| \| \| **Multivariate analysis** \| \| \| \| --- \| --- \| --- \| --- \| --- \| --- \| --- \| \| **HR***^1^* \| **95% CI***^1^* \| **P value** \| **HR***^1^* \| **95% CI***^1^* \| **P value** \| \| Age \| 1.03 \| 1.02-1.05 \| **< 0.001** \| 1.01 \| 1.00-1.03 \| **0.031** \| \| Gender \| 0.94 \| 0.72-1.24 \| 0.7 \|  \|  \|  \| \| Grade \|  \|  \|  \|  \|  \|  \| \| WHO II \| - \| - \|  \| - \| - \|  \| \| WHO III \| 3.5 \| 2.29-5.35 \| **< 0.001** \| 3.2 \| 2.08-4.92 \| **< 0.001** \| \| WHO IV \| 8.9 \| 6.00-13.2 \| **< 0.001** \| 5.66 \| 3.67-8.73 \| **< 0.001** \| \| IDH status \| 2.82 \| 2.14-3.72 \| **< 0.001** \| 0.77 \| 0.54-1.08 \| 0.13 \| \| 1p/19q status \| 6.92 \| 4.07-11.8 \| **< 0.001** \| 4.67 \| 2.66-8.18 \| **< 0.001** \| \| MGMT promoter status \| 1.21 \| 0.92-1.58 \| 0.2 \|  \|  \|  \| \| Riskscore \| 3.68 \| 2.66-5.10 \| **< 0.001** \| 1.73 \| 1.19-2.51 \| **0.004** \| \| *^1^*HR = Hazard Ratio, CI = Confidence Interval \| \| \| \| \| \| \| |
| --- | --- | --- | --- | --- | --- | --- | --- | --- | --- | --- | --- | --- | --- | --- | --- | --- | --- | --- | --- | --- | --- | --- | --- | --- | --- | --- | --- | --- | --- | --- | --- | --- | --- | --- | --- | --- | --- | --- | --- | --- | --- | --- | --- | --- | --- | --- | --- | --- | --- | --- | --- | --- | --- | --- | --- | --- | --- | --- | --- | --- | --- | --- | --- | --- | --- | --- | --- | --- | --- | --- | --- | --- | --- | --- | --- | --- | --- | --- | --- | --- | --- | --- | --- | --- | --- | --- | --- | --- | --- | --- |

**Supplement Table 7. The details of candidate drugs in gliomas**

| **Name** | **Synonym** | **Experiment evidence** | **Clinical status** | **Description** |
| --- | --- | --- | --- | --- |
| Roscovitine | S1153, seliciclib | Present | No | CDK inhibitor |
| Dasatinib | BMS-354825, S1021 | Present | Phase II | BCR-ABL kinase inhibitor |
| yostatin.1 | Not Available | Present | No | PKC inhibitor |
| CGP.60474 | Not Available | Absent | No | CDK inhibitor |
| BMS.536924 | S1012 | Present | No | IGF-1 inhibitor |
| PHA.665752 | S1070 | Present | No | c-Met inhibitor |
| RDEA119 | Refametinib | Absent | No | MEK inhibitor |
| PD.0325901 | Mirdametinib, S1036 | Present | Phase I/II | MEK inhibitor |
| Rapamycin | sirolimus, S1039, AY-22989 | Present | Phase II | MTOR inhibitor |
| XMD8.85 | ERK5-IN-1 | Absent | No | ERK inhibitor |
| CGP.082996 | Not Available | Absent | No | CDK inhibitor |
| JW.7.52.1 | Not Available | Absent | No | MTOR inhibitor |

## Supplementary Figures

**
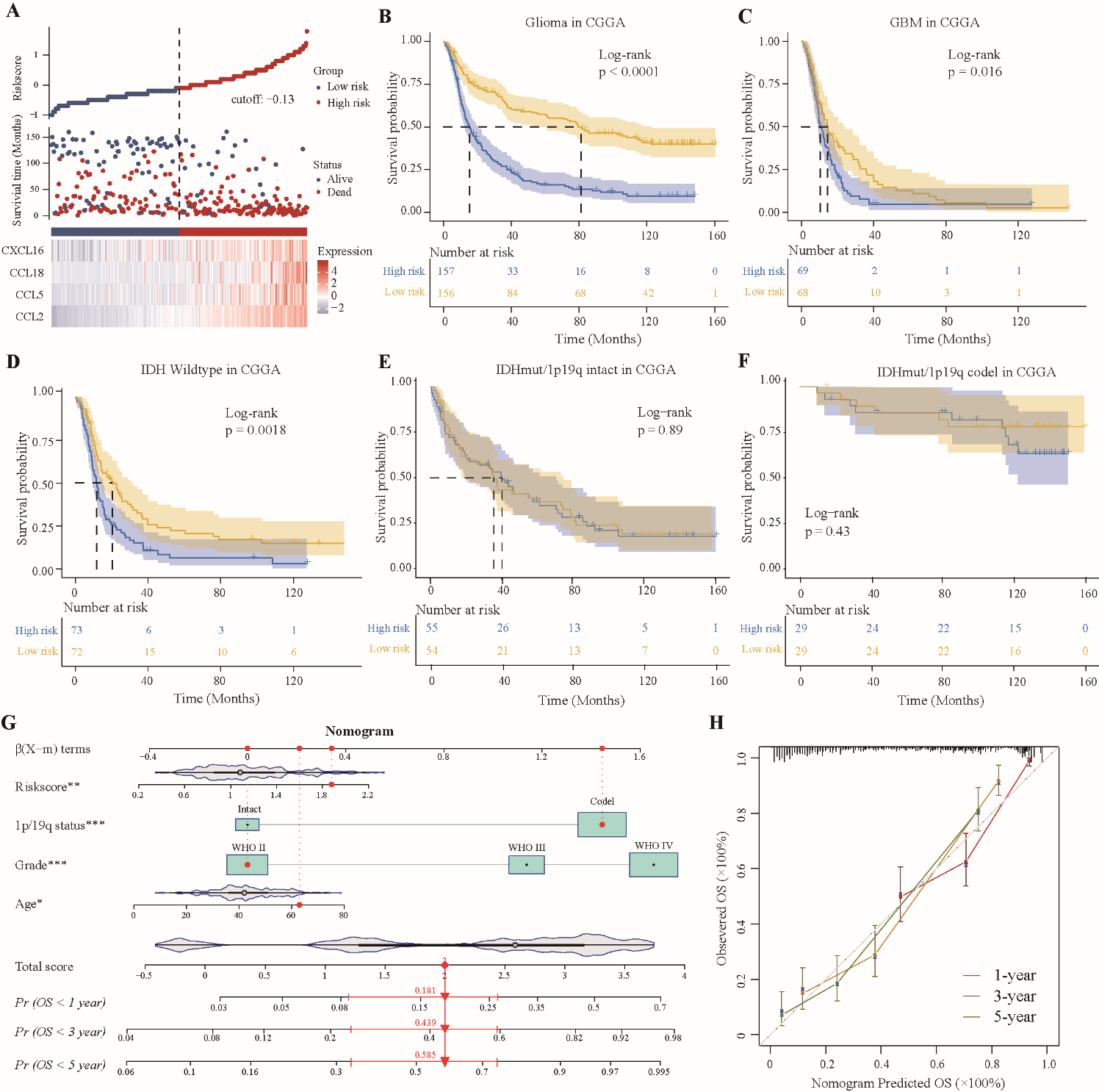
**

**Supplement Fig 1. KM survival analysis validation in CCGA dataset and nomogram for survival prediction**. A, The 4-chemokine based signature risk score distribution, the vital status of patients in the high-risk and low-risk groups and the expression profiles of related genes. B-F, Kaplan-Meier survival curves were plotted to estimate the overall survival probabilities for the low-risk versus high-risk group in all grade’s gliomas (B), GBM (C), *IDH* wildtype (D), *IDH*mut/1p19q intact (E) and *IDH*mut/1p19q codel (F) (log-rank test). G, The nomogram prediction of glioma patients for 1-, 3-, and 5-year OS combining the signatures with clinicopathological features. H, Calibration curves used to compare the predicted nomogram and measured survival probabilities in CGGA dataset, the dashed diagonal line represents the ideal nomogram.


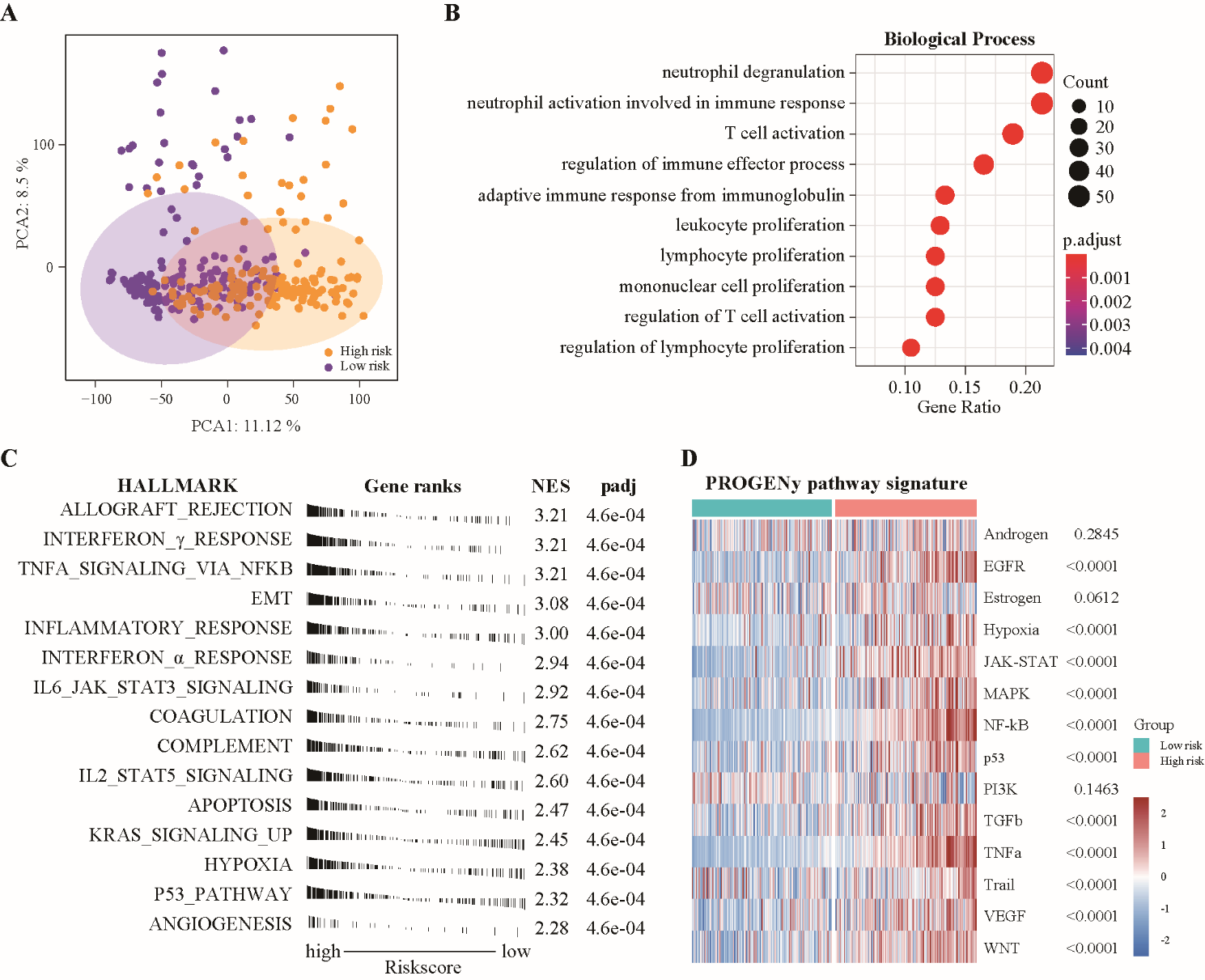


**Supplement Fig 2. Biological processes and signal pathways associated with the 4-chemokine signature in CGGA dataset.** A, Correlation between chemokines-based prognostic signature and transcriptomic expression proﬁles. B, Biological processes enrichment of positively associated genes with riskscore in CGGA dataset. C, Enriched gene sets in HALLMARK collection by GSEA in CGGA dataset. D, Heatmap of signaling pathway activity scores between high- and low-risk group by PROGENy. (Wilcoxon test)


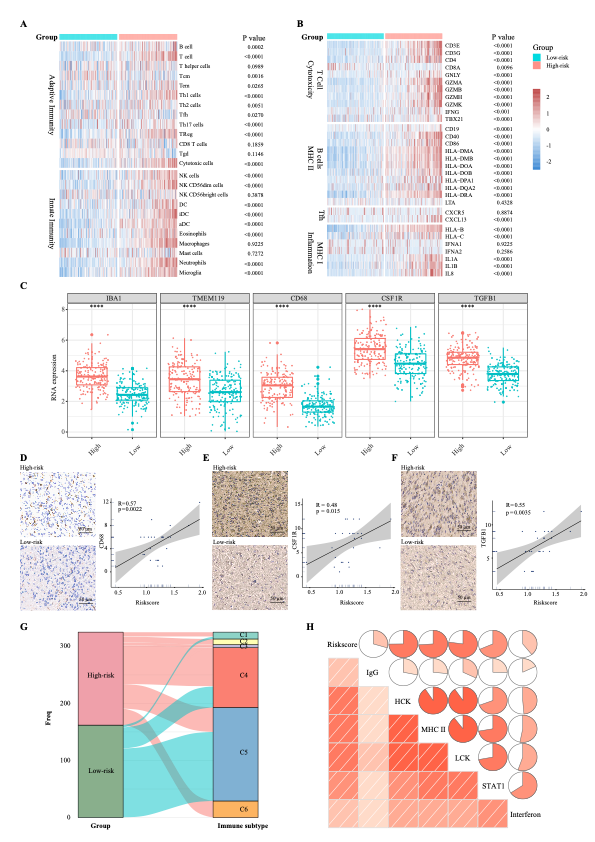


**Supplement Fig 3. Immune cell inﬁltration and inﬂammatory proﬁles of the signature in CGGA dataset.** A, Heatmap of adaptive and innate immune cell types in high- and low-risk group. B, Heatmap of the MHC-, costimulation-, and inﬂammatory-related genes expression in glioma patients from high- and low-risk group. C, The representative GAM related gene expression level between high- and low-risk group. D-F, The representative IHC images and the correlation plot between riskscore and protein expression of (D) CD68. (E) CSF1R and (F) TGFB1. G, Sankey plot shows the relationship between glioma patients stratified by riskscore and 6 immune subtypes defiend by Thorsson *et al*. H, The relationship between riskscore and inﬂammatory activity in glioma. (Wilcoxon test)
